# Supplementary material for: Mechanisms governing the pioneering and redistribution capabilities of the non-classical pioneer PU.1
Source: Nat Commun. 2020 Jan 21;11:402. doi: 10.1038/s41467-019-13960-2 (PMC6972792; doi:10.1038/s41467-019-13960-2)
Supplement: Supplementary file 7 — Source data [file 41467_2019_13960_MOESM7_ESM.zip › Source_Data/FigureS2/FigureS2B_MotifScanOutput/KmeansCluster5/homerResults/motif45.similar.html]

motif45

## Information for motif45

C
T
G
A
A
C
G
T
C
T
G
A
C
T
A
G
T
A
C
G
C
G
T
A
G
A
C
T
C
T
A
G
G
A
C
T
C
A
T
G
A
G
T
C
  
Reverse Opposite:  

T
C
A
G
G
T
A
C
C
T
G
A
A
G
T
C
C
T
G
A
G
C
A
T
A
T
G
C
G
A
T
C
G
A
C
T
T
G
C
A
G
A
C
T
  

|  |  |
| --- | --- |
| p-value: | 1e-22 |
| log p-value: | -5.184e+01 |
| Information Content per bp: | 1.675 |
| Number of Target Sequences with motif | 75.0 |
| Percentage of Target Sequences with motif | 1.88% |
| Number of Background Sequences with motif | 171.2 |
| Percentage of Background Sequences with motif | 0.40% |
| Average Position of motif in Targets | 96.7 +/- 49.4bp |
| Average Position of motif in Background | 96.4 +/- 65.5bp |
| Strand Bias (log2 ratio + to - strand density) | 0.0 |
| Multiplicity (# of sites on avg that occur together) | 1.00 |
| Motif File: | file (matrix) reverse opposite |

### Similar de novo motifs found

|  |  |  |  |  |  |  |  |
| --- | --- | --- | --- | --- | --- | --- | --- |
| Rank | Match Score | Redundant Motif | P-value | log P-value | % of Targets | % of Background | Motif file |
| 1 | 0.814 | C G A T T C A G C T A G A C G T C G T A C G T A C T A G T C G A G A T C C T A G A C G T A C T G G T A C | 1e-20 | -47.382783 | 0.80% | 0.05% | motif file (matrix) |
| 2 | 0.799 | A T C G C A G T C G T A C T G A C T A G G T C A G A T C C T A G A C G T A C T G T G A C G C A T | 1e-17 | -40.298435 | 0.85% | 0.09% | motif file (matrix) |
